# Virtual Reality to Improve Pain Management and Mental Health in Stroke Survivors With Chronic Pain: Study Protocol for a Feasibility Randomized Controlled Trial on Virtual Reality-Acceptance and Commitment Therapy

**DOI:** 10.2196/80611

**Published:** 2026-02-06

**Authors:** Sérgio A Carvalho, Paulo Menezes, Catarina Duarte, David Skvarc, Ana Rita Sousa e Silva, Ana Valentim, João Emanuel Diogo, João Sargento-Freitas, Inês A Trindade, Paula Castilho, Teresa Lapa, Gerhard Andersson, Miguel Castelo-Branco

**Affiliations:** 1 University of Coimbra, Center for Research in Neuropsychology and Cognitive and Behavioral Intervention (CINEICC), Rua do Colégio Novo, s/n, Coimbra, 3000-115, Portugal, 351 239851450 ext 375; 2 Institute of Systems and Robotics (ISR), Faculty of Sciences and Technology, University of Coimbra, Coimbra, Portugal; 3 Coimbra Institute for Biomedical Imaging and Translational Research (CIBIT), Institute of Nuclear Sciences Applied to Health (ICNAS), University of Coimbra, Coimbra, Portugal; 4 School of Psychology, Faculty of Health, Deakin University, Victoria, Australia; 5 Department of Anesthesiology, Hospitais da Universidade de Coimbra, Coimbra, Portugal; 6 Center for Classical and Humanistic Studies (CECH), Coimbra, Portugal; 7 Department of Neurology, Universidade de Coimbra Faculdade de Medicina, Coimbra, Portugal; 8 EMBRACE Lab, Center for Health and Medical Psychology, School of Behavioural, Social and Legal Sciences, University of Örebro, Orebro, Sweden; 9 Faculty of Health Sciences, Universidade da Beira Interior, Covilhã, Portugal; 10 Department of Behavioural Sciences and Learning, Linköping University, Linköping, Sweden; 11 Department of Biomedical and Clinical Sciences, Linköping University, Linköping, Sweden

**Keywords:** chronic poststroke pain, virtual reality, acceptance and commitment therapy, feasibility, mixed method, study protocol

## Abstract

**Background:**

Studies suggest that 40% to 65% of stroke survivors develop chronic poststroke pain (CPSP), which severely affects their quality of life and mental health. Empirical evidence suggests that existing treatments often fall short, underscoring the need for innovative, integrative interventions. Virtual reality (VR) seems to provide valuable tools in stroke rehabilitation. Also, contextual-behavioral psychological approaches, such as acceptance and commitment therapy (ACT), offer promising pain management and mental health resources, which seem to be feasible in VR formats. However, their combined application in CPSP remains unexplored.

**Objective:**

This study protocol describes the VR-ACT study, which will test the feasibility and preliminary efficacy of an 8-week VR-ACT program for CPSP.

**Methods:**

This pilot randomized controlled trial (N=30) will compare a VR-based ACT intervention with a sham VR control. The study will follow a mixed methods approach. Quantitative outcomes include pain intensity, psychological symptoms, and quality of life (via self-report measures), and brain network connectivity of the Triple Network (via functional magnetic resonance imaging). Feasibility will be evaluated through adherence, engagement, and acceptability. Qualitative feedback will be collected postintervention.

**Results:**

This study was funded by the Portuguese Foundation for Science and Technology in February 2025. Data collection is expected to start in December 2025 and end in June 2026. Results are expected to be published in the fall/winter of 2026/2027.

**Conclusions:**

This trial is expected to support the hypothesis that a VR-delivered ACT program is a feasible, acceptable, and potentially effective tool to support pain self-management and mental health in patients with CPSP, thereby laying the groundwork for larger multicenter trials.

## Introduction

Chronic poststroke pain (CPSP: pain lasting for more than 3 mo after stroke) affects 40% to 65% of stroke survivors [1] and can develop within 6 months after stroke [2]. CPSP substantially impacts patients’ quality of life, increases the likelihood of functional dependence and cognitive decline, and increases the risk of depression, anxiety, and fatigue [3]; it is also a predictor of suicidality after stroke [4]. Pain is experienced up to 10 years after stroke [5], prevents optimal participation and gains during rehabilitation, and patients respond poorly to medication [6]. The etiology of CPSP appears to result from an interplay of psychosocial factors (eg, mental health, coping, and social support) [7] and neurophysiological factors (eg, stroke-related damage in corticospinothalamic pathways) [8], suggesting a biopsychosocial etiology. This seems to echo the hyperconnectivity of cognitive emotional brain networks (eg, default mode network [DMN], salience network [SN], and frontal parietal network [FPN]) in chronic pain [9]. Overall, pain after stroke is underresearched [3] and is perceived by patients as an unmet need [10]. Also, health care providers report organizational barriers (eg, insufficient human resources, infrastructure, training) to implementing stroke rehabilitation guidelines [11], which calls for the development of innovative, cost-effective pain management interventions for stroke survivors [12].

Virtual reality (VR) encompasses immersive human-digital interaction technologies [13]. VR seems to be effective in pain management [14] and a viable way to avoid the detrimental effects of opioid-centered analgesia plans [15]. VR technologies can enhance the efficacy of established behavioral interventions by standardizing the presentation of images, instructions, and environmental sounds that accompany behavioral exercises and by increasing the accessibility of practice (eg, patients can use it at home, at their own pace) [16]. VR also seems to be a feasible delivery method of meditation-focused behavioral interventions, which promote a decrease of pain sensitivity by decoupling executive and pain-relevant brain regions (eg, somatosensory cortex) [17]. Additionally, VR poststroke behavioral interventions appear to enhance rehabilitation compliance due to the diverse virtual experiences offered during therapy and the potential for gamification [18]. However, its effectiveness remains inconclusive in terms of clinical validity, as most studies focus on experimental pain [19] and/or fail to adhere to sound randomized controlled trial standards (eg, lack of placebo-controlled studies) [20]. Also, the integration of self-report psychological processes and neurophysiological assessment is still incipient in VR’s efficacy studies in pain management [21].

Acceptance and commitment therapy (ACT) is a third-wave cognitive behavioral approach that promotes awareness and acceptance of internal experiences (eg, thoughts, emotions, physical sensations), and engagement with valued activities, through mindfulness and behavioral exercises [22]. ACT is especially helpful in managing chronic health conditions, as its primary focus is not on changing symptoms, but rather on promoting acceptance of difficult internal experiences and making behavioral changes towards a valued and meaningful life. Stroke survivors find ACT helpful in promoting acceptance and adjustment to stroke limitations, in fostering a sense of safety, and in developing self-regulatory skills [23]. ACT seems to lead to reductions in the triple network connectivity involved in pain chronicity, namely brain networks related to self-reflection (DMN), emotion (SN), and cognitive control (FPN) [24]. Although ACT is feasible and effective in reducing psychopathology in stroke survivors [25], its feasibility in CPSP management is yet to be explored. Additionally, although ACT has been found effective in pain management using technological formats (eg, online platforms, mobile apps) [26,27], ACT through VR has not been extensively tested. One recent study suggests ACT through VR is feasible and effective in improving mental health [28]. However, most VR interventions have been almost exclusively focused on exposure techniques or breathing meditation practices [29], rather than full psychological programs that promote emotional self-regulation skills (eg, attentional regulation) and reduction of avoidant-focused behavior (eg, commitment to valued action), and lack methodologically sound designs (eg, placebo-controlled).

## Methods

### Study Objectives

This project has 2 objectives: (1) we aimed to develop and test the feasibility of an 8-week VR-ACT program in a sample of patients with CPSP. By “feasibility,” we mean feasibility in practice, that is, the extent to which the VR-ACT program can be delivered, measured by *retention* and *acceptability* of the recruitment and intervention implementation phases [30]. (2) We aimed to pilot test the efficacy of VR-ACT in improving pain, mental health, and adaptive psychological processes and skills and in reducing the functional connectivity of the triple network (DMN, SN, and FPN).

### Design

**Figure 1.**
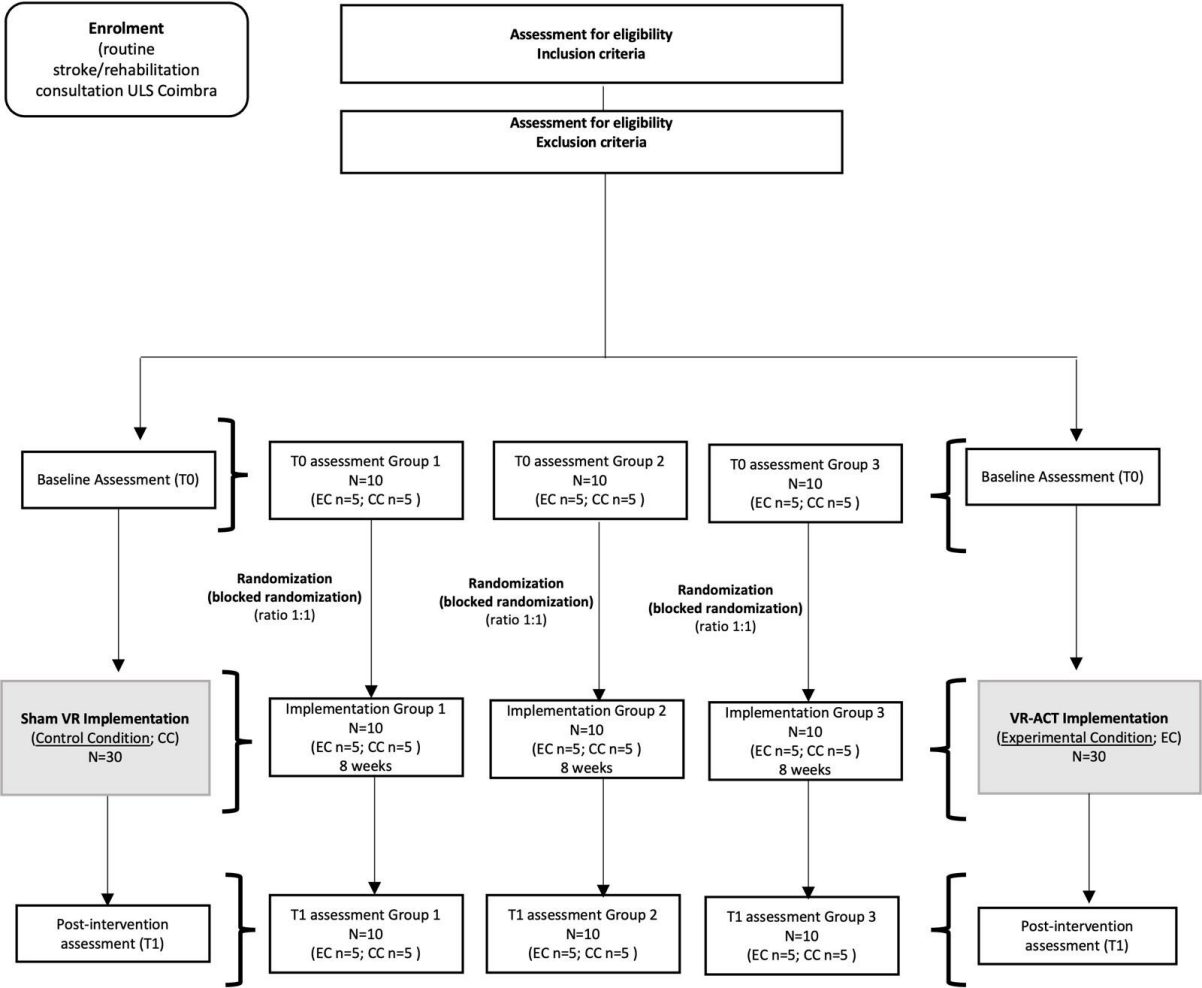
Flowchart of participants. ACT: acceptance and commitment therapy; CC: control condition; EC: experimental condition; VR: virtual reality.

** **The current project will conduct a parallel-group randomized controlled trial design and will follow a multimethod approach (qualitative and quantitative assessments) to assess the feasibility of the VR-ACT delivery [31]. The experimental condition (EC): the VR-ACT program: 8 modules of 3D immersive skills training (2 targeting awareness regulation skills; 3 promoting acceptance; 3 focused on behavioral change and commitment to valued action). To assess the preliminary efficacy and control for possible placebo effects, the control condition (CC) will consist of 8 nonimmersive 2D distracting video animations delivered through a VR headset (Sham VR). Participants in both conditions will use the VR devices throughout 8 weeks. Each module will be available weekly in both conditions and have a duration of 20 minutes. Participants in the CC will be able to use the VR-ACT after the final assessment (see Figure 1).

 During the development of the VR-ACT intervention, a patient and public involvement group will be formed to monitor the adaptation of the exercises into VR and their tailoring for patients with CPSP. The group (n=5) will consist of 1 medical doctor and 1 nurse working in CPSP rehabilitation, 1 clinical psychologist specializing in contextual behavioral approaches to chronic pain, and 2 patients with CPSP (1 male and 1 female). The research team and the patient and public involvement group will gather monthly during the development of the VR-ACT intervention to ensure the patient-centered adaptation of exercises is successful and acceptable.

### Participants

#### Eligibility Assessment

 The study will be conducted in adults diagnosed with CPSP who attend the Neurology Service, Pain Unit, and/or the Rehabilitation Service at the ULS-Coimbra, Portugal. Inclusion criteria include (1) medical diagnosis of CPSP conducted by a medical doctor collaborating with the research team, (2) age between 18 and 80 years, (3) implicit de facto internet and computer literacy, and (4) willingness to comply with the study procedures. Exclusion criteria include (1) currently with active malignancy, (2) severe cognitive impairment, (3) currently undergoing any psychological intervention or VR-delivered pain management program, (4) current severe psychiatric symptoms (ie, psychosis, severe depression, nonsuicidal self-injury, suicide attempt in the last month), (5) language impairment with severe comprehension deficit, (6) history of photosensitive epilepsy or previous experience of severe simulator sickness, and (7) other neurological conditions (eg, dementia or Parkinson disease).

#### Sample Size Calculation

 The study was powered for a large final timepoint between-group effect size (standardized mean difference=0.8), allowing for a variant autoregressive ρ over 2 measurements (preintervention and postintervention; 1 and 0.8) to accommodate the likely instability of the outcome variable(s). Setting the α at .05 and a minimum of 80% power, a minimum sample size of N=30 is required (EC: n=15; CC: n=15). We used the power.mrmm function from the R “longpower” package, through WebPower. A sample size of 15 to 30 participants is also sufficient to attain saturation in qualitative analyses conducted through interviews [32].

 To minimize dropout, (1) 2 phone calls will occur during the VR-ACT implementation to address questions on the VR-ACT content. (2) We also expect minor technical difficulties when using the VR headset. At the beginning of each module, an avatar therapist will provide a welcome message, reminding you of the research team’s contact information in case of any technical problems. Before the implementation, an online group meeting will be conducted to introduce the VR headset to participants in both conditions and help with navigation. (3) To promote engagement, weekly emails will be sent to all participants reminding them to use the VR headsets. Also, after the VR system registers 3 days of absence, automatic reminders will be sent to the participants' emails.

### Procedures

#### VR Program Development 

The VR-ACT intervention content will adapt ACT-based interventions for chronic pain that were previously developed and tested by the research team (eg, the face-to-face COMP.ACT program [33] and the online iACTwithPain platform [34]) into a VR format and tailored to CPSP. The research team will adapt experiential practices (eg, mindfulness meditation) and exercises (eg, values clarification and commitment to valued action) into a VR format through VR headset equipment (MetaQuest). The VR-ACT content will be developed through an “Unreal Engine” application with multiple rooms and different designs/environments, allowing the user-patient/participant to explore those that best suit their current need (eg, pain alleviation; emotional regulation training; establishing valued-based goals for the day) and/or the exercise suggested by the VR system (each module will have a suggested sequence; see Textbox 1).

An embedded registration system will be developed to monitor the progress and number of complete or incomplete sessions and exercises, which will be indicators of usability. This will enable the assessment of preferred time of usage and other user-centered information that can be further utilized in VR-ACT patient-centered tailoring. This registration will be supported by a centralized database and allocated to a server within the University of Coimbra, Portugal. Simultaneously, the research team will develop the content and format of the Sham VR control condition, which will be delivered through the same VR headset equipment, but with different content: 8 distraction-based exercises delivered via 2D nonimmersive video animations.

Textbox 1. **The VR-ACT intervention content.**

**Modules 1 and 2: Awareness Regulation Skills**

- Welcome to VR-ACT: control as the problem, and not the solution (Avatar Therapist)
- Introduction to Mindfulness (Avatar Therapist)
- Mindful breathing practice
- Body-scan practice
- Soothing Rhythm Breathing practice
- Mindfulness of thoughts practice (Leaves on a Stream).

**Modules 3, 4, and 5: Acceptance of Pain and Internal Experiences**

- Introduction to willingness and acceptance: Why accept? (Avatar Therapist)
- Letting go of control—part I: the quicksand metaphor (adapted to immersive VR)
- Letting go of control—part II: Joe the bum metaphor (adapted to immersive VR)
- Mindfulness of difficult emotions practice
- Accepting with self-compassion: soften, soothe, and allow practice.

**Modules 6, 7, and 8: Behavior Change and Valued Action**

- Introduction to Values (Avatar Therapist)
- The passengers on the bus metaphor (adapted to immersive VR)
- Mindfulness meditation on values: rediscovering what matters to me.
- Interactive game: 4 steps to committed action
- 90th birthday imagery practice (adapted to immersive VR)
- Life after VR-ACT: awareness and acceptance practice as building blocks of valued action.

#### Data Collection

**Figure 2.**
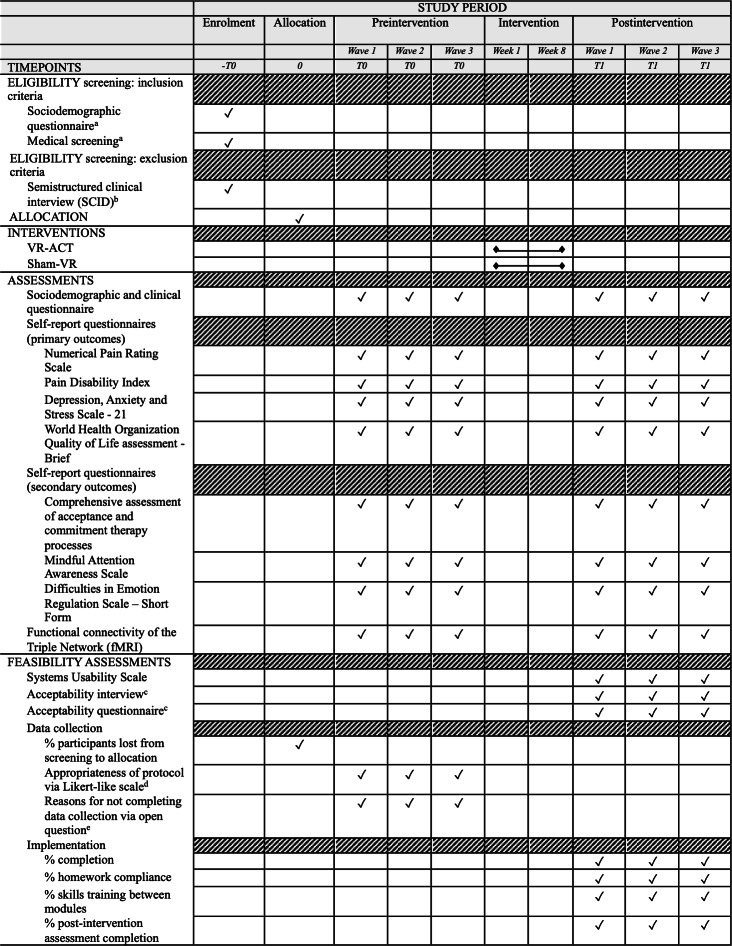
SPIRIT figure of the VR-ACT study. ^a^Conducted by the medical and nursing collaborators of the research team. ^b^Conducted by a clinical psychologist member of the research team. ^c^See Section A in Multimedia Appendix 1. ^d^From 0=not at all appropriate to 4=very much appropriate. ^e^Please let us know why you decided not to complete the data collection.

The research team members, who are physicians at the Neurology Service, the Pain Unit, and/or the Rehabilitation Unit of ULS-Coimbra, Portugal, will invite participants and inform them of the aims and conditions for participation. Those who provide written informed consent will be screened for medical eligibility. Then, psychological eligibility will be individually assessed, online and at a time preferred by participants, by the clinical psychologists of the research team, for severe neurocognitive and psychiatric symptoms (using Mini Mental State Examination and a semistructured clinical interview adapted from Structured Clinical Interview for DSM). Those who meet criteria for severe psychiatric disorders and/or cognitive impairment will be excluded from the study, given feedback, and advised to seek medical/psychological consultation. Eligible participants will receive the link to the online preintervention assessment (T0) self-report protocol. A task-oriented (see Section B in Multimedia Appendix 1) functional magnetic resonance imaging (fMRI) will be scheduled to collect preintervention data on the functional connectivity of the triple network (DMN, SN, and FPN). Participants will then be randomly assigned by a member of the research team to 1 of 2 conditions (EC and CC) using random permuted blocks to ensure balance in age (via an online randomizer, eg, random.org [35]). Participants will be informed of the starting date via telephone (or alternatively via email). The medical/nursing collaborators and the research team members not involved with the implementation monitoring will be blinded to the allocation. The two conditions will be masked to participants by not explicitly informing whether the content of the VR is 3D (VR-ACT) or 2D (Sham VR), with both being delivered through the same model of VR headset (MetaQuest 3.0). Nevertheless, for ethical reasons, the two conditions in the study are described in the patient information sheet. A scheduled meeting in the ULS-Coimbra will be held to provide headset devices to participants. Three groups or waves of recruitment and VR-ACT implementations will be conducted (group 1, n=10; group 2, n=10; group 3, n=10; see Figure 1). At the end of implementation, participants will return the devices and will conduct the post-intervention assessment (T1): online psychosocial self-report data collection; and functional connectivity of the triple network data collection (fMRI). Additionally, participants in the EC will be interviewed online by a research team member who is blind to the allocation to collect qualitative data on the acceptability and usability of the VR-ACT (see Section A in Multimedia Appendix 1). The time points of measurements are described in the SPIRIT figure (See Figure 2).

#### Feasibility Assessment

##### Assessment of Data Collection and Randomization

The feasibility [36] of the recruitment and data collection will be assessed via (1) the number of participants lost from screening to randomization; (2) *appropriateness* of size, format, and content of the assessment protocol, by responding to a Likert-like scale (from 0=not at all appropriate to 4=very much appropriate) regarding (a) *accessibility* to the protocol, (b) *willingness* to provide the required information, and (c) *size* of the protocol; (3) reasons for not completing data collection (open question “Please, let us know why you decided not to complete the data collection”); (4) support provided by medical/nursing collaborators in referring potential participants measured by average number per week of participants referred by the medical/nursing team.

##### Assessment of the VR-ACT Delivery

The feasibility of the VR-ACT implementation will be evaluated in terms of (1) *adherence*: (a) percentage of participants who completed the intervention, (b) number of sessions completed, and (c) an open-ended question asking the reasons for not completing the intervention; (2) *engagement*: (a) percentage of participants who completed homework assignments and frequency of skills practice between sessions and (b) participants’ average number of VR-ACT device log-ins; (3) *retention/attrition*: number of participants who completed the postintervention assessment; (4) *acceptability*: participants will use a Likert-like scale (eg, 0=not at all; 4=very useful) to rate the VR-ACT modules in terms of its *usefulness*, *comprehensibility*, and *likelihood to continue using learned skills*. Participants will provide suggestions to improve the content of sessions via open-ended questions (see Section A in Multimedia Appendix 1).

### Instruments

#### Demographic and Clinical Data

*** ***Demographic data will be collected through a self-report questionnaire (ie, age, marital status, education, sex assigned at birth, gender identity), as well as clinically relevant data (ie, date of stroke, date of pain onset after stroke, poststroke pain diagnosis, current medication, and other chronic pain diagnoses).

#### Primary Outcomes

##### Pain Intensity (Numeric Rating Scale)

The Numerical Pain Rating Scale [37,38] is an 11-point scale used to measure pain intensity, ranging from 0 (“No pain”) to 10 (“Worst imaginable pain”). It assesses pain through 3 ratings: current pain, highest pain in the last 24 hours, and lowest pain in the last 24 hours, which are then averaged to provide a single score of pain intensity.

##### Pain Impact (Pain Disability Index)

 The pain disability index [39] is an 11-point scale (0=“no disability”; 10=“worst disability”), a widely used scale that measures pain disability in 7 daily life domains. Higher scores indicate higher pain disability.

##### Psychological Distress (Depression, Anxiety, and Stress Scale)

 The Depression, Anxiety, and Stress Scale (DASS-21) [39,40] is a 21-item questionnaire composed of 3 subscales designed to measure the negative emotional states of depression, anxiety, and stress. Each of the 3 DASS subscales comprises seven items, which respondents rate on a 4-point severity/frequency scale (0 to 3) in relation to the extent to which they experienced each state over the past week.

##### Quality of Life (World Health Organization Quality of Life-Bref)

 The World Health Organization Quality of Life-Bref [41] is a 26-item multidimensional measure of quality of life that assesses subjective quality of life. This instrument measures four dimensions of quality of life (physical, psychological, social relations, and environment). It can also be used as a measure of general quality of life.

### Secondary Outcomes

#### Psychological (In)flexibility (Comprehensive Assessment of ACT)

 The Comprehensive Assessment of ACT [42,43] is a measure of psychological flexibility as conceptualized by ACT, with three subscales: openness to experience, valued action, and behavioral awareness. This project will use the short version with 18 items, measured in a Likert-like 7-point scale (0–“Strongly disagree” to 6–“Strongly agree”).

#### Emotional (Dys)regulation (Difficulties in Emotion Regulation Scale-Short Form)

 The Difficulties in Emotion Regulation Scale-Short Form (DERS-SF) [44,45] is an 18-item version of the DERS scale that assesses difficulties in regulating emotions in 6 different dimensions, 3 items per dimension, measured in a 5-point Likert scale (1=almost never to 5=almost always): nonacceptance of emotional responses, (2) difficulties engaging in goal-directed behavior, (3) impulse control difficulties, (4) lack of emotional awareness, (5) limited access to emotion regulation strategies, and (6) lack of emotional clarity. This scale can be used unidimensionally to measure difficulties in emotional regulation, where higher scores mean more difficulties in emotional regulation.

#### Mindful Awareness (Mindful Attention Awareness Scale)

 The Mindful Attention Awareness Scale [46,47] is a 15-item scale designed to assess a core characteristic of mindfulness, namely, a receptive state of mind in which attention, informed by a sensitive awareness of what is occurring in the present, simply observes what is taking place. It measures mindful awareness in a 6-point Likert-like scale (1=almost always; 6=almost never).

#### The Triple Network

 The functional connectivity of the Triple Network will be assessed through fMRI, specifically examining the functional connectivity of brain regions involved in chronic pain, including brain networks related to self-reflection (DMN), emotion (SN), and cognitive control (FPN).

#### System Usability Scale

 The System Usability Scale [46,47] is a 10-item scale that measures global view system usability (example item: “I thought the system was easy to use”), through a 5-point response scale ranging from 1=strongly disagree to 5=strongly agree.

### Data Analyses: Feasibility and Preliminary Efficacy

The feasibility of VR-ACT will be assessed qualitatively through open-ended questions (eg, “What would you say were the benefits of using the VR-ACT headset?,” “What have you learned from the VR-ACT program?”) designed to explore adherence, engagement, and acceptability of the intervention (see Section A in Multimedia Appendix 1). Thematic analyses will be conducted with the support of NVivo, and common themes in each question will be categorized. Also, feasibility will be quantitatively assessed through (1) dropout rate, (2) number of usage of the VR headset (embedded software usage monitoring), (3) percentage of intervention completion, (4) a 5-point Likert scale measuring usefulness, comprehensibility, and likelihood to continue using learned skills. The usability of the VR-ACT will be quantitatively assessed through the System Usability Scale.

The preliminary efficacy of VR-ACT will be pilot tested through 2 statistical methods: (1) intention to treat analyses, using linear mixed models to examine statistically significant change over time, moderated by allocation, and post hoc differences, adjusted for multiple comparisons using the Bonferroni technique; (2) reliable change index for each outcome for preintervention to postintervention timepoints, to assess clinically significant individual changes [48]. Network-based statistics [49] will be used to assess significant differences in network connectivity within and between conditions.

### Ethical Considerations

All procedures will be conducted in accordance with the 1964 Helsinki Declaration and its subsequent amendments or comparable ethical standards. This study was approved by the Ethics Committee of the Faculty of Psychology and Educational Sciences at the University of Coimbra (ref: CEDI/FPCEUC: 98/R-1). The study will be conducted in accordance with European directives for research involving human participants, such as the General Data Protection Regulation. All participants will provide informed consent to participate in the study. Participants will be provided a patient information sheet with the following information: (1) the aim of the study and the eligibility criteria for participating; (2) the different phases of the study, including assessments and the randomization; (3) the benefits and risks of participating (namely that “We do not anticipate any risks arising from your participation in the study. However, if participating in the study results in a negative emotional experience and you need support, please contact the research team” and other national health resources will be provided). In this case, their participation will be interrupted. (4) Participation is voluntary, participants have the right to drop out without being obligated to provide justification, and access to treatment as usual, as well as their relationships with the medical and nursing team, will not be influenced by this decision; also, the decision to participate will not interfere with their medical treatment; (5) the confidentiality of data will be ensured in all results dissemination activities, and identifiable data in datasets will be password protected, to which only the research team will have access; (6) audio recording of postintervention interviews will be immediately deleted after written transcription. Informed consent will be obtained from each participant by the medical/nursing collaborator who will invite patients to participate before any data collection. (7) The current study will not conduct an assessment of neurological pathology; thus, data collected through fMRI will not be indicative of clinical diagnoses nor associated pathophysiology. The study results will be provided to interested participants in a format that is accessible.

A steering committee composed of 1 participant, 1 physician (anesthesiologist), 1 clinical psychologist, and 1 ethicist will monitor the delivery of the 8-week VR-ACT intervention and ensure the ethical and scientific integrity of the study (eg, adherence to the protocol). The steering committee will gather online at weeks 1, 3, 6, and 8 to discuss the trial progress, methodological issues arising from the implementation, and ethical decision-making. The trial will be interrupted if >50% of participants in the VR-ACT condition report increased psychological distress after session 1, and/or if >50% of the members of the steering committee decide so.

## Results

This study was funded by the Portuguese Foundation for Science and Technology in February 2025 (2023.13402.PEX; [50]). Data collection is expected to start in December 2025 and end in June 2026. Data analysis will start in June 2026 and continue until September 2026. Results are expected to be disseminated via a peer-reviewed paper prepared and published in the fall/winter of 2026/2027. The research team has not started recruitment. Recruitment will start on December 1, 2025, and end on January 31, 2026. See Section D in Multimedia Appendix 1 for Trial registration—data set. The VR-ACT project intends to disseminate its results to 3 targeted audiences: (1) the scientific community, via peer-reviewed empirical studies with the results of its feasibility and preliminary efficacy study; (2) the clinical/professional community (eg, health professionals delivering care to patients with CPSP, through oral and poster presentations in scientific/professional conferences, and scientific meetings with health care services; (3) the general community, via social media, mainstream media. The authorship in all scientific outputs will be determined according to the authors’ contributions and the recommendations of the International Committee of Medical Journal Editors [51].

## Discussion

### Expected Scientific Contribution of VR-ACT

This protocol outlines the feasibility and preliminary efficacy study of the VR-ACT project. The project adopts a multidisciplinary approach by integrating scientific knowledge from medicine, neuroscience, psychology, and computer science and engineering to develop an ACT-based VR self-management program that aims to help patients with CPSP improve their pain management skills and mental health. It employs a mixed methods approach, measuring feasibility and preliminary efficacy through both quantitative and qualitative methodologies. It is expected that (1) the implementation of VR-ACT will be feasible, measured by adherence (ie, attrition rate), engagement (number of VR usage; completion), and acceptability (qualitative postintervention assessment); (2) the VR-ACT program will be significantly more effective in reducing pain intensity and disability, and psychopathological symptoms, and in increasing quality of life, as well as in reducing functional connectivity of the triple network (fMRI), than the Sham VR.

This study is expected to have potential scientific and societal contributions: (1) development of an innovative cost-effective evidence-based pain management and mental health resource for patients with CPSP that integrates recent evidence on protective factors in CP (eg, acceptance-based emotional regulation skills, and attentional redirection) using technologies with potential personal and societal economic sustainability, thus being in alignment with the 2030 agenda for sustainable development of the United Nations (eg, access to physical and mental health and well-being for all); (2) a multimethod approach to feasibility and efficacy, by integrating qualitative and quantitative data, as well as both psychological and neuroimaging data, which will add to the literature a multilevel understanding of (preliminary) efficacy. The project will potentially open a new avenue of research in CPSP (ie, the development of self-regulatory skills through digital solutions in CPSP rehabilitation) and tentatively reinforce the need for biopsychosocial multimodal approaches to CPSP to be included in the training of health professionals working with CPSP. These expected results on the feasibility and preliminary efficacy of VR-ACT in CPSP will serve as a reference point for a future, larger, multicenter, long-term, cross-cultural study.

The current study has some potential limitations that should be considered. Due to its feasibility focus, the study is powered for a small sample size (N=30), which limits the statistical power to detect small effect sizes and also restricts the ability to draw definitive conclusions regarding the efficacy of the VR-ACT intervention. Additionally, although an effort will be made to control for the placebo effect with a sham-VR condition, controlling for expectancy and placebo effects in VR interventions remains challenging, and it can be anticipated that participants may still distinguish between conditions despite our efforts to mask the condition allocated. Finally, the generalizability of the expected results can be hindered by strict eligibility criteria. For example, the inherent technological literacy required to participate, as well as the absence of severe psychological and neurological disorders, may limit the applicability of the results to the broader CPSP population.

### Conclusions

This study protocol provides information on the implementation and feasibility testing of the VR-ACT project, specifically the development, feasibility, and preliminary efficacy testing of an ACT intervention delivered via VR, tailored to enhance the pain management skills and mental health of patients with CPSP. This is an early-stage trial that has the potential to serve as a reference point for future larger-scale effectiveness studies of VR-ACT and related resources to improve pain self-management and mental health of patients with CPSP.

## Supplementary material

10.2196/80611 Multimedia Appendix 1 VR-ACT post-intervention semi-structured interview; mindfulness task-based fMRI; information about the study; all items from the World Health Organization Trial Registration Data Set.

10.2196/80611 Peer Review Report 1 Peer review report by Fundação para a Ciência e a Tecnologia (FCT), Portugal.

## Abbreviations

ACT: acceptance and commitment therapy
CC: control condition
CPSP: chronic poststroke pain
DASS: Depression, Anxiety and Stress Scale
DERS-SF: Difficulties in Emotion Regulation Scale-Short Form
DMN: default mode network
EC: experimental condition
fMRI: functional magnetic resonance imaging
FPN: frontal parietal network
SN: salience network
VR: virtual reality
