# Supplementary material for: Virtual Reality to Improve Pain Management and Mental Health in Stroke Survivors With Chronic Pain: Study Protocol for a Feasibility Randomized Controlled Trial on Virtual Reality-Acceptance and Commitment Therapy
Source: JMIR Res Protoc. 2026 Feb 6;15:e80611. doi: 10.2196/80611 (PMC12902207; doi:10.2196/80611)
Supplement: Multimedia Appendix 1 [file resprot-v15-e80611-s001.docx]

**APPENDICES**

**Section A**

**VR-ACT Post-intervention semi-structured interview**

| ***1. How would you describe your experience with VR-ACT?*** |
| --- |

| ***2. What would you say were the benefits of using the VR-ACT headset?*** |
| --- |

| ***3. What have you learned from the VR-ACT program?*** |
| --- |

| ***4. Which module/theme did you find most useful? In what way was it useful?*** |
| --- |

| ***5. How often did you practice the skills in your daily life (i.e., when not using the VR-ACT headset)?*** |
| --- |

| ***6. What was most difficult to grasp, to understand, and/or to implement during the 8-week VR-ACT program?*** |
| --- |

| ***7. What was your experience with the research team whenever you needed help with the VR-ACT intervention.*** |
| --- |

| ***8. Do you have any suggestions to improve the VR-ACT and/or its implementation?*** |
| --- |

**Quantitative assessment of the perceived usefulness and impact of VR-ACT**

**(Self-report)**

**1. After participating in the VR-ACT program, I feel my pain is:**

| 1  (worse) | 2  (the same) | 3  (improved) | 4  (improved significantly) |
| --- | --- | --- | --- |

**2. Since I started VR-ACT, the way I cope with my pain is:**

| 1  (worse) | 2  (the same) | 3  (improved) | 4  (improved significantly) |
| --- | --- | --- | --- |

**3. Since I started VR-ACT, the way I cope with my overall difficulties is:**

| 1  (worse) | 2  (the same) | 3  (improved) | 4  (improved significantly) |
| --- | --- | --- | --- |

**4. How much would you be interested in continuing using the VR-ACT headset:**

| 1  (nota at all) | 2  (possibilly) | 3  (interested) | 4  (very much interested) |
| --- | --- | --- | --- |

**5. How useful did you find the skills promoted by the VR-ACT:**

| 1  (not al all) | 2  (a little) | 3  (useful) | 4  (very useful) |
| --- | --- | --- | --- |

**6. How difficult did you find practicing the exercises:**

| 1  (very difficult) | 2  (difficult) | 3  (easy) | 4  (very easy) |
| --- | --- | --- | --- |

**7. To what extent did you use the skills learned with the VR-ACT in your daily life:**

| 1  (never) | 2  (rarely) | 3  (sometimes) | 4  (many times) |
| --- | --- | --- | --- |

Section B

Mindfulness task-based fMRI


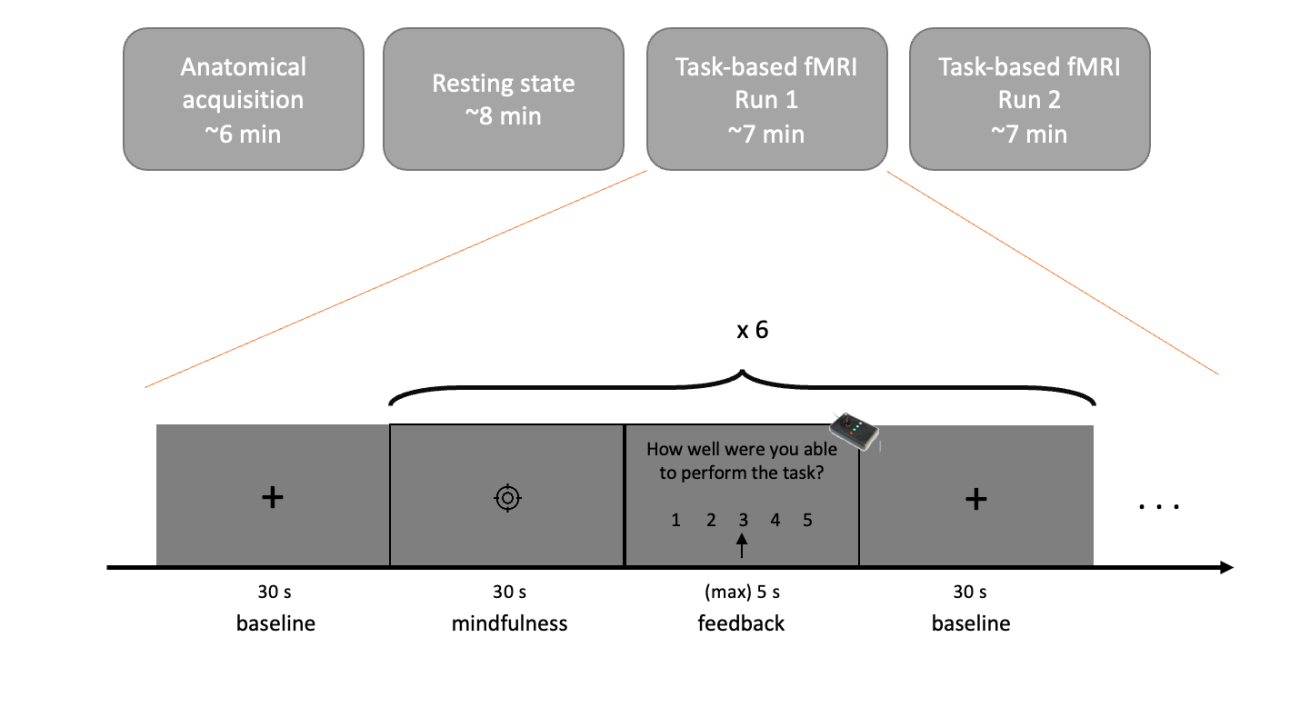


**Section C**

**INFORMAÇÃO SOBRE O ESTUDO**

**O ESTUDO**

A presente investigação, com título “*VR-ACT após AVC: estudo RCT da viabilidade da ACT em Realidade Virtual para promoção de gestão da dor e saúde mental em pessoas com Dor*

*Crónica Pós-AVC*”, a decorrer na Faculdade de Psicologia e Ciências da Educação da Universidade de Coimbra (FPCEUC), e financiado pela Fundação para a Ciência e Tecnologia (FCT; 2023.13402.PEX). O presente estudo tem como objetivo desenvolver e testar a viabilidade de uma intervenção psicológica para a gestão da dor e promoção de saúde mental através de realidade virtual (VR-ACT) em pessoas com dor crónica pós-AVC. Este estudo foi submetido a consulta pela Comissão de Ética e Deontologia da Investigação da Faculdade de Psicologia e Ciências da Educação da Universidade de Coimbra (CEDI-FPCEUC), no âmbito das suas competências, tendo obtido parecer favorável.

**A SUA PARTICIPAÇÃO**

A participação no presente estudo tem três fases:

Fase 1: Antes do programa VR-ACT

Antes de iniciar o programa, a equipa de investigação (um membro da equipa da área da medicina, e um membro da equipa da área da psicologia) analisará a sua elegibilidade* para participar no presente estudo. A sua elegibilidade será analisada através de uma breve entrevista (aproximadamente 30-40 minutos) através de plataforma online (e.g., Zoom), numa data e horário de acordo com a sua disponibilidade. Se não cumprir os critérios de elegibilidade, ser-lhe-á explicado por que razão não poderá participar no estudo. Se cumprir os critérios de elegibilidade, receberá no seu email uma ligação para um protocolo online de questionários de autorresposta que deverá preencher (tempo de preenchimento aproximado de 40 minutos). Após preenchimento, será contactado/a por email para agendar a recolha de dados de conexão funcional da Rede Tripla por ressonância magnética funcional (fMRI). Esta recolha será realizada presencialmente no Centro de Imagem Biomédica e Investigação Translacional (CIBIT), e terá a duração aproximada de 30 minutos. Durante a recolha de dados por fMRI, ser-lhe-á pedido que realize um breve exercício de meditação guiada por instruções áudio. Esta recolha de dados por fMRI não tem uma natureza clínica, pelo que alterações morfológicas, anatómicas, ou funcionais de natureza clinicamente relevante não serão alvo de análise clínica. Contudo, na eventualidade de serem identificadas alterações morfológicas, anatómicas, ou funcionais com possível relevância clínica, essa informação ser-lhe-á fornecida. Todos os dados recolhidos por fMRI ser-lhe-ão fornecidos.

Fase 2: Durante o programa VR-ACT

Após a análise da elegibilidade para participar, assim como a recolha de dados, será aleatoriamente alocado/a a uma de duas condições possíveis, através de um software informático: condição 1) VR-ACT: nesta condição, as pessoas participantes terão acesso ao programa VR-ACT. O programa VR-ACT é composto por 8 módulos integrados num dispositivo de realidade virtual, através do qual exercícios de meditação, psicoeducação e comportamentais serão disponibilizados; condição 2) VR-Sham: nesta condição, as pessoas participantes terão acesso, durante 8 semanas, a conteúdos de animação de caráter distrativo. Cada módulo terá a duração de aproximadamente 20 minutos, com a possibilidade de repetir os conteúdos e práticas sempre que assim o desejar durante as 8 semanas. No final do programa, caso tenha sido alocado/a à condição VR-Sham, ser-lhe-á dada oportunidade de realizar o VR-ACT, caso o deseje. A equipa de investigação fornecer-lhe-á os dispositivos de realidade virtual. No final das 8 semanas, deverá devolver esses dispositivos à equipa.

Fase 3: Após o programa VR-ACT

Após o término do programa, receberá um email com uma ligação para um protocolo online de questionários de autorresposta que deverá preencher (tempo de preenchimento aproximado de 40 minutos), assim como será agendada uma entrevista online (e.g., Zoom) para que possa partilhar connosco a sua experiência com o programa. O áudio da entrevista online será gravado e posteriormente transcrito para um documento (e.g., Word). Será ainda agendada uma última recolha de dados por fMRI, semelhante à realizada na Fase 1.

*Posso participar? (elegibilidade)

Para **poder participar** no estudo, deverá cumprir os seguintes critérios:

a) diagnóstico médico de dor crónica pós-AVC; b) idade entre 18 e 80 anos.

**Não poderá participar** neste estudo **se**: a) atualmente tiver uma doença oncológica em fase ativa; b) estiver atualmente a ser acompanhado/a em consultas de psicologia; c) tiver atualmente sintomatologia psiquiátrica severa (e.g., depressão severa, psicose, ideação suicida durante o último mês); d) historial de epilepsia fotossensível; e) outras condições ou doenças neurológicas (e.g., Demência, Parkinson, declínio cognitivo).

**O QUE BENEFICIO EM PARTICIPAR DESTE ESTUDO?**

Ao participar no estudo, contribuirá para o conhecimento científico sobre novas modalidades e/ou formatos de intervenção psicológica na promoção de gestão da dor e saúde mental em pessoas com dor crónica pós-AVC. Para além disso, terá acesso a um recurso de realidade virtual que, a partir da evidência empírica sobre os seus princípios subjacentes, esperamos poder ajudá-lo/a a lidar de forma mais eficaz com a sua dor, assim como melhor a sua saúde mental.

**PARTICIPAÇÃO VOLUNTÁRIA**

A sua participação é inteiramente voluntária, tendo o direito de recusar participar se assim o entender. A qualquer momento da sua participação pode decidir interromper a sua colaboração, sem que daí decorram quaisquer consequências. Após a sua participação, pode, a qualquer momento, se assim o entender, e sem ter de fornecer qualquer justificação, retirar o seu consentimento, bastando enviar um email ao investigador responsável (sergiocarvalho@fpce.uc.pt), sendo as suas respostas eliminadas do estudo. A sua decisão de participar ou não no presente estudo não interfere com o seu tratamento habitual.

**CONFIDENCIALIDADE DOS DADOS**

Os resultados obtidos são estritamente confidenciais e serão utilizados apenas para fins da presente investigação. A sua participação no estudo não implicará quaisquer danos para si, tendo apenas de despender de aproximadamente 40 minutos no preenchimento do protocolo online. Os custos da sua participação consistem nas 2 deslocações às instalações do CIBIT para recolha de dados por fMRI. Os seus dados individuais manter-se-ão confidenciais e anonimizados em todas as bases de dados, de acordo com os regulamentos e leis aplicáveis. A base de dados estará protegida por uma palavra-passe a que apenas a equipa de investigação terá acesso. Os seus dados serão eliminados das bases de dados 5 anos após a sua participação no estudo. O áudio gravado na entrevista após a intervenção (Fase 3) será imediatamente eliminado após o término do estudo, permanecendo apenas a transcrição escrita, que será eliminada 5 anos após o término do estudo. As dados recolhidos por fMRI serão guardados nos laboratórios do CIBIT, anonimizados, e protegidos por palavra-passe a que apenas a equipa de investigação terá acesso, e destruído 5 anos após o término do estudo. Em todas as atividades de divulgação científica, os seus dados individuais nunca serão divulgados, sendo os resultados divulgados unicamente de forma

agregada (ou seja, no total de todas as pessoas participantes), e não de forma individualizada, e de forma inteiramente anonimizada.

**COMPROMISSO**

A equipa de investigação compromete-se a:

a) Garantir a total confidencialidade dos dados fornecidos.

b) Utilizar os dados fornecidos estritamente para fins da presente investigação.

c) Garantir o anonimato dos dados em qualquer atividade de divulgação dos resultados.

c) Prestar esclarecimentos sobre quaisquer dúvidas acerca da investigação, em qualquer momento.

**RISCO ASSOCIADO À PARTICIPAÇÃO**

Não antecipamos quaisquer riscos decorrentes da sua participação no estudo. No entanto, e ainda que os conteúdos da intervenção serem de baixo ou médio caráter imersivo e de baixa intensidade sensorial, o caráter imersivo da utilização de tecnologia de realidade virtual poderá, em algumas pessoas, contribuir para a experiência de algum desconforto pelo caráter multissensorial de alguns exercícios do programa. Se em algum momento sentir desconforto, sensorial ou de outro tipo, decorrente do uso do dispositivo de realidade virtual (por exemplo, stress, tonturas, frustração), entre em contacto com a equipa de investigação ([sergiocarvalho@fpce.uc.pt](mailto:sergiocarvalho@fpce.uc.pt)). Poderá ainda contactar a linha Saúde 24 – 808 24 24 24.

A presente investigação não irá proceder à avaliação da presença de patologia neurológica, pelo que os dados recolhidos não serão nunca informativos de eventuais diagnósticos ou patofisiologia associada.

**COMPENSAÇÃO PELA PARTICIPAÇÃO NO ESTUDO**

A equipa de investigação ou a Universidade de Coimbra não se responsabilizarão, financeiramente ou de outra forma, com os custos das deslocações ao CIBIT. Contudo, a equipa de investigação disponibilizará um seguro para a eventualidade de acidente decorrido durante deslocação ao CIBIT.

Caso queira obter informação sobre os resultados do estudo, envie um email para o investigador responsável, Sérgio A. Carvalho (sergiocarvalho@fpce.uc.pt), solicitando os resultados globais do estudo. Um breve relatório dos resultados do estudo ser-lhe-á enviado através de correio eletrónico.

**O INVESTIGADOR RESPONSÁVEL**

Sérgio A. Carvalho

sergiocarvalho@fpce.uc.pt

239 851 450

CINEICC, Universidade de Coimbra

**A EQUIPA DE INVESTIGAÇÃO**

Sérgio A. Carvalho, CINEICC, FPCEUC [Investigador Responsável]

Ana Rita Esteves de Sousa e Silva, CINEICC, FPCEUC

Ana do Rosário Caleiro Valentim, Serviço de Anestesiologia, CHUC

David Skvarc, Deakin University

Isabel Catarina Duarte, Centro de Imagem Biomédica e Investigação Translacional (CIBIT)

João Emanuel Fernandes Serra Rodrigues Diogo, FLUC

João André Sargento Araújo De Freitas, Serviço de Neurologia, CHUC

Maria Inês Oliveira e Costa de Almeida Trindade, Orebro University

Paula Cristina de Oliveira de Castilho Freitas, CINEICC, FPCEUC

Paulo Jorge Carvalho Menezes, Instituto de Sistemas e Robótica

Teresa Lapa, Serviço de Anestesiologia, CHUC

Miguel de Sá e Sousa de Castelo Branco, CIBIT [consultor]

Gerhard Andersson, Linköping University [consultor]

**CONSENTIMENTO INFORMADO**

Declaro ter compreendido os objetivos do estudo e que a minha participação no mesmo consiste no preenchimento de questionários de autorresposta (duração aproximada de 40 minutos) em 2 momentos de avaliação (antes da intervenção e após a intervenção), assim como na recolha de dados através de ressonância magnética. Foi-me informado que o presente estudo não fará uma análise patológica nem diagnóstica dos dados recolhidos, pelo que a equipa de investigação não terá conhecimento da presença de patologia neurológica ou de outro tipo associada. Foi-me garantida a possibilidade de recusar participar neste estudo, a qualquer momento, sem qualquer tipo de consequências. Foi-me garantido um seguro contra acidentes decorridos na deslocação decorrentes da participação no estudo. Declaro que me foi garantida a confidencialidade dos meus dados e que as minhas respostas serão utilizadas apenas para fins desta investigação, sem qualquer informação individualizada e/ou que me possa identificar.

Sim ________ Não _________

**[ENGLISH TRANSLATION]**

**PATIENT INFORMATION SHEET**

**INFORMED CONSENT**

**THE STUDY**
This research, entitled “VR-ACT after Stroke: RCT Study on the Feasibility of ACT in Virtual Reality for Promoting Pain Management and Mental Health in People with Post-Stroke Chronic Pain”, is being conducted at the Faculty of Psychology and Educational Sciences of the University of Coimbra (FPCEUC) and is funded by the Portuguese Foundation for Science and Technology (FCT; 2023.13402.PEX). The aim of this study is to develop and assess the feasibility of a psychological intervention using virtual reality (VR-ACT) to promote pain management and mental health in individuals with post-stroke chronic pain. This study has been reviewed and approved by the Ethics and Research Deontology Committee of FPCEUC (CEDI-FPCEUC).

**YOUR PARTICIPATION**
Participation in this study consists of three phases:

**Phase 1: Before the VR-ACT Program**
Before starting the program, the research team (comprising a member from the medical field and a member from psychology) will assess your eligibility to participate in this study. This assessment will involve a short interview (approximately 30–40 minutes) conducted via an online platform (e.g., Zoom), scheduled at a time that suits your availability. If you do not meet the eligibility criteria, you will be informed of the reasons for exclusion. If you are eligible, you will receive an email with a link to an online self-report questionnaire protocol to be completed (approximately 40 minutes).
After completing the questionnaire, you will be contacted via email to schedule a functional connectivity data collection session of the Triple Network using functional magnetic resonance imaging (fMRI). This session will take place in person at the Centre for Biomedical Imaging and Translational Research (CIBIT) and will last approximately 30 minutes. During this session, you will be asked to perform a brief guided meditation exercise with audio instructions. Please note that this fMRI scan is non-clinical, and clinically relevant morphological, anatomical, or functional alterations will not be the focus of analysis. However, if any potentially clinically significant findings are observed, you will be informed. All fMRI data collected will be made available to you.

**Phase 2: During the VR-ACT Program**
After confirming your eligibility and completing the baseline data collection, you will be randomly assigned to one of two possible conditions using computer software:

1. **VR-ACT** – Participants in this condition will access the VR-ACT program, which includes 8 modules within a virtual reality headset. These modules offer meditation, psychoeducation, and behavioral exercises.
2. **VR-Sham** – Participants in this condition will view 8 weeks of distraction-based animated content. Each module lasts approximately 20 minutes and may be repeated at your discretion during the 8-week period.

At the end of the program, participants who were in the VR-Sham group will be offered the opportunity to experience the VR-ACT program, if they wish. The research team will provide all necessary virtual reality equipment, which must be returned at the end of the 8 weeks.

**Phase 3: After the VR-ACT Program**
After completing the program, you will receive an email with a link to another online self-report questionnaire (approximate completion time: 40 minutes). You will also be scheduled for an online interview (e.g., Zoom) to share your experience with the program. The audio of this interview will be recorded and transcribed (e.g., into a Word document).
Finally, a second fMRI session, similar to the one in Phase 1, will be scheduled.

**Am I eligible?**
You may participate in this study if you meet the following criteria:
a) Medical diagnosis of post-stroke chronic pain;
b) Aged between 18 and 80 years.

You will **not** be eligible if you:
a) Have active cancer;
b) Are currently undergoing psychological treatment;
c) Exhibit severe psychiatric symptoms (e.g., severe depression, psychosis, suicidal ideation in the last month);
d) Have a history of photosensitive epilepsy;
e) Have other neurological conditions (e.g., dementia, Parkinson’s disease, cognitive decline).

**WHAT DO I GAIN BY PARTICIPATING?**
By participating in this study, you will contribute to scientific knowledge about new psychological intervention formats for promoting pain management and mental health in individuals with post-stroke chronic pain. Additionally, you will gain access to a virtual reality resource that, based on existing evidence, may help you manage your pain more effectively and improve your mental health.

**VOLUNTARY PARTICIPATION**
Your participation is entirely voluntary. You have the right to decline participation or withdraw at any time without consequence. You may also withdraw your consent at any

point after participating, without needing to provide any justification. Simply send an email to the lead researcher (sergiocarvalho@fpce.uc.pt), and your data will be removed from the study. Your decision to participate or not will have no effect on your usual medical care.

**DATA CONFIDENTIALITY**
All collected data will remain strictly confidential and will be used solely for the purposes of this research. Your participation will not involve any harm, and the only time commitment required is the 40 minutes to complete the online protocol. The only costs to you will be travel to and from the CIBIT center for the two fMRI sessions. Your personal data will be anonymized and stored securely, with access restricted to the research team. All data will be deleted 5 years after your participation. Audio recordings from the post-intervention interview (Phase 3) will be deleted after the study ends, with the transcript retained for 5 years. fMRI data will be stored at CIBIT, anonymized, and securely protected, and will also be destroyed 5 years after study completion. No individual data will ever be published—only aggregate results will be reported.

**RESEARCH TEAM COMMITMENTS**
The research team commits to:
a) Ensuring complete confidentiality of the data provided;
b) Using the data solely for research purposes;
c) Guaranteeing anonymity in all dissemination activities;
d) Providing clarification at any point during the research process.

**RISKS ASSOCIATED WITH PARTICIPATION**
We do not anticipate any risks from participation. However, while the VR content is of low to moderate intensity, the immersive nature of virtual reality may cause mild discomfort in some individuals due to the multisensory experiences. If you experience discomfort (e.g., stress, dizziness, frustration), please contact the research team (sergiocarvalho@fpce.uc.pt) or the Portuguese Health Line (Saúde 24) at 808 24 24 24.
This study will not evaluate neurological conditions, and any data collected cannot be used for clinical diagnosis.

**COMPENSATION**
The research team and the University of Coimbra will not reimburse travel costs to the CIBIT center. However, insurance coverage will be provided in case of accidents during travel to CIBIT.
If you wish to receive a summary of the study’s results, please email the lead researcher, Sérgio A. Carvalho (sergiocarvalho@fpce.uc.pt). A brief report of the findings will be sent to you via email.

**LEAD RESEARCHER**
**Sérgio A. Carvalho**
sergiocarvalho@fpce.uc.pt
+351 239 851 450
CINEICC, University of Coimbra

**THE RESEARCH TEAM**

Sérgio A. Carvalho, CINEICC, FPCEUC [Investigador Responsável]

Ana Rita Esteves de Sousa e Silva, CINEICC, FPCEUC

Ana do Rosário Caleiro Valentim, Serviço de Anestesiologia, CHUC

David Skvarc, Deakin University

Isabel Catarina Duarte, Centro de Imagem Biomédica e Investigação Translacional (CIBIT)

João Emanuel Fernandes Serra Rodrigues Diogo, FLUC

João André Sargento Araújo De Freitas, Serviço de Neurologia, CHUC

Maria Inês Oliveira e Costa de Almeida Trindade, Orebro University

Paula Cristina de Oliveira de Castilho Freitas, CINEICC, FPCEUC

Paulo Jorge Carvalho Menezes, Instituto de Sistemas e Robótica

Teresa Lapa, Serviço de Anestesiologia, CHUC

Miguel de Sá e Sousa de Castelo Branco, CIBIT [consultant]

Gerhard Andersson, Linköping University [Consultant]

**INFORMED CONSENT**

I declare that I have understood the objectives of the study and that my participation involves completing self-report questionnaires (approximately 40 minutes) at two assessment points (before and after the intervention), as well as participating in data collection through magnetic resonance imaging (fMRI). I have been informed that this study will not perform any pathological or diagnostic analysis of the data collected, and therefore, the research team will not be aware of the presence of any neurological or other associated conditions. I have been assured that I may refuse to participate in this study at any time, without any consequences. I have also been guaranteed accident insurance coverage for travel related to participation in the study. I declare that the confidentiality of my data has been guaranteed and that my responses will be used solely for the purposes of this research, with no individualized or personally identifying information being disclosed.

Yes _____ No ______

**Section D**

| **All items from the World Health Organization Trial Registration Data Set** | |
| --- | --- |
| **Data category** | **Information** |
| Primary registry and trial identifying number | ClinicalTrials.gov  NCT06990646 |
| Date of registration in primary registry | 21 June 2025 |
| Secondary identifying numbers | CEDI FPCEUC Ethics Committee  CEDI/FPCEUC:98/R-1 |
| Source(s) of monetary or material support | Fundação para a Ciência e a Tecnologia, I.P.  2023.13402.PEX  2021.01871.CEECIND |
| Primary sponsor | Fundação para a Ciência e a Tecnologia, I.P. |
| Secondary sponsor(s) | Not appliacable. |
| Contact for public queries | Project Manager  Carla Cardoso [ccardoso@uc.pt] |
| Contact for scientific queries | Sérgio A. Carvalho [sergiocarvalho@fpce.uc.pt]  Center for Research in Neuropsychology and Cognitive and Behavioral Intervention (CINEICC) |
| Public title | VR-ACT after stroke: a feasibility study |
| Scientific title | VR-ACT after Stroke: a feasibility RCT of a Virtual Reality delivery of ACT for pain management and mental health in patients with Chronic Post-Stroke Pain |
| Countries of recruitment | Portugal |
| Health condition(s) or problem(s) studied | Chronic Post-Stroke Pain |
| Intervention(s) | Active comparator: VR-ACT (Acceptance and Commitment Therapy via virtual reality) |
|  | Placebo comparator: Sham-VR (2D non-immersive distracting video-animations delivered through a VR headset) |
| Key inclusion and exclusion criteria | Inclusion: a) medical diagnosis of CPSP conducted by a medical doctor collaborating with the research team; b) age between 18 and 80; c) implicit de facto internet and computer literacy; d) willingness to comply with the study procedures |
|  | Exclusion: a) currently with active malignancy; b) severe cognitive impairment; c) currently undergoing any psychological intervention or VR-delivered pain management program; d) current severe psychiatric symptoms (i.e., psychosis; severe depression; non-suicidal self-injury; suicide attempt in the last month); e) language impairment with severe comprehension deficit; f) history of photosensitive epilepsy or previous experience of severe simulator sickness; g) other neurological conditions (e.g., dementia; Parkinson´s) |
| Study type | Interventional |
|  | Allocation: random permuted blocks (1:1) |
|  | Primary purpose: feasibility assessment |
|  | Phase I-II |
| Date of first enrolment | January 2026 |
| Target sample size | N = 30 |
| Recruitment status | Not yet recruiting |
| Primary outcome(s) | Feasibility assessment:  1) *adherence*: a) percentage of participants who completed the intervention; b) number of sessions completed; c) an open-ended question asking the reasons for not completing the intervention; 2) *engagement*: a) percentage of participants who completed homework assignments and frequency of skills practice between sessions; b) participants´ average number of VR-ACT device log-ins; 3) *retention/attrition*: number of participants who completed the post-intervention assessment; 4) *acceptability*: Participants will use a Liker-like scale (0 = not at all; 4 = very much) to rate the VR-ACT modules in terms of its *usefulness*, *comprehensibility*, *likelihood to continue using learned skills*  Preliminary efficacy assessment:  Pain intensity, Pain disability, Psychopathological symptoms (depression, anxiety, stress), Quality of life. |
| Key secondary outcomes | Psychological (In)flexibility, Mindful awareness, Difficulties in emotion regulation. |
|  | |
