# Supplementary material for: Virtual Reality to Improve Pain Management and Mental Health in Stroke Survivors With Chronic Pain: Study Protocol for a Feasibility Randomized Controlled Trial on Virtual Reality-Acceptance and Commitment Therapy
Source: JMIR Res Protoc. 2026 Feb 6;15:e80611. doi: 10.2196/80611 (PMC12902207; doi:10.2196/80611)
Supplement: Peer Review Report 1 [file resprot-v15-e80611-s002.pdf]

2023.13402.PEX

# Evaluation Report

RESEARCHER
SÉRGIO CARVALHO

TITLE
VR-ACT after Stroke: a feasibility RCT ...

WEIGHT 20%

## A1 – Scientific merit of the project

SCORE
8,00

**Comment**

This project aims to develop and pilot test, in a randomized controlled trial (RCT) design with a sham VR as control condition, the feasibility, acceptability and preliminary efficacy of a self-management VR 8-week program for pain management and mental health in patients with Chronic Post Stroke Pain. As intervention, an Acceptance and Committee Therapy based program will be used. The project is of great interest. It starts from a real point of view, pointing out that the study of CPSP and its treatment has yet to be demonstrated, given the small number of studies and their characteristics. However, it is true that psychological and multidisciplinary treatment is the most appropriate option. The choice of ACT as a psychological treatment is appropriate. Although CBT is the treatment of choice for chronic pain, considering ACT is a wise move: it has its roots in CBT and provides aspects that have proven useful in recent years, especially in chronic pain. It is, therefore, an option of interest when adding knowledge in an area that requires research. The age of inclusion is wide (from 18-80), but it can be expected that particularly the younger group will be underrepresented given the fact that a stroke more often occurs in older (or middle aged) individuals. The study is introduced in a coherent way, and most aspects of the design, methods and procedures, including a power analysis and statistical analysis section, are well-described and contain enough detail. However, more detail would be needed on the control condition, in which distraction tasks may reduce pain.
Overall, this looks like a straightforward and interesting trial, albeit that it might be somewhat ambitious to run the trial in a relatively short period of time.

**Score**
8,00

WEIGHT 20%

## A2 – Innovative nature of the proposal

SCORE
7,50

**Comment**

The new aspect of this study is the application of a ACT based program in VR for pain management, which represents a highly desirable multidisciplinary intervention in this type of problem. Whereas ACT has been shown to be promising for different outcomes, such as mental health, there is little evidence that it can be used to reduce pain, particularly through a VR tool. In that respect, the project is novel, although it builds on previous research. The integration of self-reported, qualitative, and quantitative data as well as physiological records is of great interest and unusual in the field, which makes the study more interesting.

What is missing is the extent to which results obtained through virtual reality are expected to generalize to the lives of participants. It focuses on pain, emotions, and quality of life, but the role of pain acceptance and recovery of activities is not expressly mentioned.

**Score**
7,50

WEIGHT 21%

## B1 – Scientific merit of the Principal Investigator and the research team

SCORE
8,00

**Comment**

The PI of this project holds a PhD in Clinical Psychology since 2021, and has worked for a year as an assistant professor with some clinical work in a private practise as well. More recently, the PI started as junior research fellow and teaches two courses. As such, the PI is just starting up their academic career after their PhD in 2021. Still the PI has been active in the academic community by (co-)supervising students, including 5 ongoing PhD students, and presenting findings at conferences, while being involved in a number of international projects as well. The PI has also contributed to the scientific field by adapting an intervention to an online platform, and validating an instrument to assess pain. The team includes a mix of more junior and senior researchers and clinicians, with complementary expertise, while two very senior consultants are included as well. Prof. Andersson, one of the experts, is an international leader in the field of pain treatment and the use of information and communication technologies to apply cognitive behavioral therapy and third generation therapies, is, therefore, a guarantee for the project.

The rest of the team is made up of doctors in psychology, medicine (anesthesiology) and engineers who cover aspects related to the recording of physiological measurements and computer scientists. All of them have extensive research and clinical experience in topics related to the project.

Part of the research team has joint publications prior to this project.

**Score**
8,00

WEIGHT 14%

## B2 – Impact of project execution for PI's career progression and/or research

SCORE
**7,50**

**Comment**

The PI has a recent research development. Doctor in 2021, he has only been participating in research projects for a few years. This project, as a PI, will allow you to expand and consolidate your research activity in an area and with an appropriate team to achieve your objectives. It will be a way to acquire the leadership that is currently lacking. Also, by the interdisciplinary nature, the knowledge of the PI will be extended and their international network will be developed.

**Score**

7,50

WEIGHT 12.5%

## C1 – Feasibility of the work plan and proposed indicators

SCORE
**6,50**

**Comment**

The task descriptions and expected results are clearly described and include a lot of details, and overall this looks like an ambitious, yet feasible work plan. The activities and timelines are included and seem to be fitting with the projects' aims. There is no contingency plan included for when the recruitment is going less well than expected or when too many participants drop out, which can be expected given their medical status as post-stroke patients. Some more publications are missing on the results of the study, considering the wide variety of primary and secondary measures used. There is also a lack of greater dissemination of the results in the clinical and social context.

**Score**

6,50

WEIGHT 12.5%

## C2 – Budget adequacy

SCORE
**8,50**

**Comment**

The budget seems well justified. It describes the acquisition of VR equipment and software licenses, needed to run the trial of course, and salary of researchers to put time and effort into task development for the VR program. Also, consultancy hours are included in the budget, which seems very reasonable. The cost of MetaQuest units is adequate, even lower than usual. In addition, other researchers' salary are budgeted, as well as scanning costs of participants. There is no detailed information on the software for the VR-ACT and Sham-VR, which in any case is low, given the global amount allocated. All in all, the budget looks ambitious but feasible.

**Score**

8,50

## Ethical issues

Ethical concerns reported in the proposal:

Humans | Protection of personal data

Did you find any ethical concerns that aren't included in the proposal? **No**

## Global Budget

Do you agree with the requested budget? **Yes**

| Items | Solicited |
|---|---|
| Human Resources | 15 134,08 € |
| Missions | 0 |
| Scientific and technical instruments and equipment | 8 400,00 € |
| Subcontracts | 0 |
| Patents registration | 0 |
| Demonstration, Promotion and Dissemination | 4 400,00 € |
| Adaptation of buildings and facilities | 0 |
| Purchase of goods and services | 12 010,00 € |
| Overheads | 9 986,02 € |
| Total | **49 930,10 €** |

## Final Score 7,71
